# DiscML: an R package for estimating evolutionary rates of discrete characters using maximum likelihood

**DOI:** 10.1186/1471-2105-15-320

**Published:** 2014-09-27

**Authors:** Tane Kim, Weilong Hao

**Affiliations:** Department of Biological Sciences, Wayne State University, 48202 Detroit, USA; Mathematics Undergraduate Program, Wayne State University, 48202 Detroit, USA

**Keywords:** Discrete character states, Gene family evolution, Birth and death, Maximum likelihood, Phylogeny

## Abstract

**Background:**

The study of discrete characters is crucial for the understanding of evolutionary processes. Even though great advances have been made in the analysis of nucleotide sequences, computer programs for non-DNA discrete characters are often dedicated to specific analyses and lack flexibility. Discrete characters often have different transition rate matrices, variable rates among sites and sometimes contain unobservable states. To obtain the ability to accurately estimate a variety of discrete characters, programs with sophisticated methodologies and flexible settings are desired.

**Results:**

DiscML performs maximum likelihood estimation for evolutionary rates of discrete characters on a provided phylogeny with the options that correct for unobservable data, rate variations, and unknown prior root probabilities from the empirical data. It gives users options to customize the instantaneous transition rate matrices, or to choose pre-determined matrices from models such as birth-and-death (BD), birth-death-and-innovation (BDI), equal rates (ER), symmetric (SYM), general time-reversible (GTR) and all rates different (ARD). Moreover, we show application examples of DiscML on gene family data and on intron presence/absence data.

**Conclusion:**

DiscML was developed as a unified R program for estimating evolutionary rates of discrete characters with no restriction on the number of character states, and with flexibility to use different transition models. DiscML is ideal for the analyses of binary (1s/0s) patterns, multi-gene families, and multistate discrete morphological characteristics.

**Electronic supplementary material:**

The online version of this article (doi:10.1186/1471-2105-15-320) contains supplementary material, which is available to authorized users.

## Background

Many evolutionary processes involve transitions among different discrete characteristic states, including changes in morphological characteristics [1], sequence gain and loss [2, 3], gene family expansion and contraction [4], gain and loss of mobile promoters [5] and epigenetic characteristics such as methylation [6]. Evolutionary rates of discrete characters have been estimated using programs primarily developed for constructing ancestral character states such as the ACE function of the APE package [7] in R, standalone programs BayesTraits [8] and Mesquite [9]. Recently, great efforts have been made to estimate gene family turnover rates. The GLOOME program maps gain and loss rates using binary characters (or 1s/0s) [10], while Count [11], BEGFE [12], BadiRate [13], and CAFE3 [14] employ birth-and-death (BD) models to study gene family expansion and contraction.

Some of these programs have advanced (or realistic) features that are not implemented in other programs. For instance, the BayesTraits program implements a *Γ*-distribution for rate variation [8]. The GLOOME program allows the estimation of prior root probabilities of the character states [10, 15]. The BadiRate program allows variable birth rates and death rates, and corrects for unobservable data [13]. Furthermore, many multistate characters do not necessarily evolve in a BD manner [16], and should therefore be modeled using transition rate matrices other than BD.

In order to perform accurate rate estimation on a variety of discrete characters, we have developed a unified program DiscML by implementing the advanced features mentioned above as well as flexible options for transition rate matrices.

## Implementation

DiscML estimates the evolutionary rates of discrete characters by fitting the distribution of all character states (the data) on a given phylogeny. The data need to be in a matrix format (vector format for a single site) as required in many other phylogenetic programs in R (see examples in Additional file 1). The provided phylogeny is required to have branch lengths, as branch lengths will be used as a relative time scale in the analysis. The evolutionary rates, transition rate matrices, and additional parameters discussed below will be optimized to maximize the likelihood of the data. The optimization is achieved using the PORT routines [17] implemented in the nlminb function in R.

### Implementation of rate variation in the analysis

Rate variation among the character sites has long been recognized and implemented in DNA analyses [18], but has been missing from most analyses of non-DNA discrete characters (but see [8]). DiscML considers rate variation among the character sites by implementing a discrete *Γ* distribution (with the option of alpha=TRUE).

### Estimation of prior root probabilities

Most programs for the analysis of discrete characters assume only uniformly distributed prior root probabilities, e.g., , (*a* is the total number of character states). DiscML allows the estimation of prior root probabilities ( *π*_*a*_) for different character states (with the option of rootprobability=TRUE).

### Flexibility on both the transition model and the number of character states

DiscML is flexible on both the size and type of the transition rate matrix (*Q*), which can be customized by users. This option could open the door for novel evolutionary analyses on different discrete characters. Several transition rate matrices are pre-determined in DiscML: model=~ER~ (equal rates, i.e., all entries in equation 1 are equal), model=~SYM~ (symmetric, i.e., *α*_1_=*α*_2_, *β*_1_=*β*_2_, *γ*_1_=*γ*_2_, ..), and model=~ARD~ (all rates different, i.e., all entries are free to vary). ER and SYM are reversible matrices, while ARD matrices are irreversible.


Evolutionary processes can follow a birth-and-death (BD) process. The birth processes correspond to transitions from state *n* to state *n*+1, while the death processes correspond to transitions from state *n* to state *n*−1. The BD transitions can be represented as matrices containing non-zero entries only between the neighboring states (equation 2). Several pre-determined BD transition rate matrices are available: BDER (equal rates), BDSYM (symmetric, i.e., *α*_1_=*α*_2_, *β*_1_=*β*_2_, *γ*_1_=*γ*_2_, ..), BDISYM (symmetric, all entries except *α* are equal, i.e., *α*_1_=*α*_2_, *β*_1_=*β*_2_=*γ*_1_=*γ*_2_=..), and BDARD (all rates different).


Finally, all transition rate matrices (*Q*s) are calibrated [19], i.e., each *Q* satisfies
3

so that the evolutionary rate parameter ( *μ*) is the average number of transition events per site per evolutionary time unit [20].

### Forced reversibility and flexible irreversible options

When the prior root probabilities ( *π*) for different character states are estimated, reversible transition matrices will no longer necessarily result in reversible evolutionary processes (because of potentially different probabilities of character states). Since it is sometimes of biological interest to assume reversibility (i.e., the expected *x*→*y* changes equal to the *y*→*x* changes), DiscML can allow forced reversibility by setting reversible=TRUE. In practice, reversibility is obtained by multiplying the corresponding root probabilities (equation 4) to the entries in reversible transition matrices, e.g., ER and SYM. Such a practice is conceptually the same with the general time-reversible (GTR) DNA substitution model [21]. In DiscML, model=~GTR~ is equivalent to the combination of model=~SYM~ and reversible=TRUE.


Similarly, when the prior root probabilities for different character states are estimated, forced reversibility can be applied to the BD related matrices (equation 5).


In DiscML, the default setting is reversible=FALSE and users have the flexibility to conduct analysis by assuming irreversible evolutionary processes. Unlike in reversible processes, the root position can greatly affect the maximum likelihood calculation in irreversible cases [22, 23]. Therefore, it is only meaningful to perform irreversible analysis on a rooted tree. If the provided phylogenetic tree is unrooted, DiscML will first reroot the tree by midpoint rooting, and perform analysis on the midpoint rooted tree.

### Correction for unobservable data

Some characters may contain unobservable character states, which can only be inferred indirectly from the presence of observable states of the same characters in related taxa. Ancient characters can be lost from all examined extant taxa, and result in unobservable data. DiscML provides the option of zerocorrection=TRUE to calculate the likelihood conditional on a pattern being observable following [24], i.e.,
6

where *L*_−_ is the likelihood of unobservable patterns. The correction for unobservable data (shown as ‘+0’ in Table 1) is essential for systems such as gene family data due to the complete loss of some ancient genes, but not suitable for single-site analyses and for systems in which all character states are observable (e.g., nucleotide bases).

**Table 1:** **DiscML estimates from the gene family data in the Bacillaceae (B1, B2, B3) clades**

Models	Parameters	B1	B2	B3
ER	*μ*	3.073	0.677	0.540
(1s/0s only)	Ln*L*	-15150	-16467	-22229
ER+0	*μ*	1.887	0.463	0.388
(1s/0s only)	Ln*L*	-13682	-15268	-21207
BDER	*μ*	2.490	0.590	0.485
	Ln*L*	-20901	-22196	-29127
BDISYM	*μ*	2.669	0.556	0.438
	Ln*L*	-19684	-20973	-27811
BDARD	*μ*	5.746	1.369	1.450
	Ln*L*	-18254	-20073	-26578
ER	*μ*	2.940	0.638	0.459
	Ln*L*	-21411	-23273	-31405
SYM	*μ*	2.635	0.546	0.427
	Ln*L*	-19615	-20947	-27801
ARD	*μ*	5.601	1.345	1.314
	Ln*L*	-18143	-19678	-26239
GTR	*μ*	3.731	0.739	0.632
(SYM+ *π* ^REV^)	Ln*L*	-17753	-19337	-25381
ER+0	*μ*	2.339	0.531	0.395
	Ln*L*	-20595	-22586	-30753
ER+ *π*	*μ*	2.935	0.624	0.454
	Ln*L*	-20070	-21783	-28771
ER+ *Γ*	*μ*	3.205	0.638	0.459
	Ln*L*	-21398	-23273	-31405
ER+0+ *π* + *Γ*	*μ*	1.358	0.236	0.240
	Ln*L*	-18719	-19960	-26712
ER+0+ *π* ^REV^ + *Γ*	*μ*	3.630	0.379	0.387
	Ln*L*	-16839	-17960	-23398

The parameter *μ* is the estimated evolutionary rate of the characters. “1s/0s only” indicates binary analysis by converting all non-zero characters to 1s using simplify=TRUE, ‘+0’ indicates the correction for unobservable data using zerocorrection=TRUE, ‘+ *Γ*’ indicates the implementation of a discrete *Γ* distribution using alpha=TRUE, ‘+ *π*’ indicates the estimation of prior root probabilities using rootprobability=TRUE, ‘+ *π* ^REV^’ indicates the estimation of prior root probabilities with forced reversibility using rootprobability=TRUE and reversible=TRUE.

### Site and branch specific estimations

Even though the default setting of DiscML is to perform rate estimation by fitting the distribution pattern of all character sites on a phylogeny, there is an option to perform rate estimation on individual sites (ind=TRUE). Individual rates can be graphically displayed using plotmu=TRUE. Furthermore, DiscML allows branch specific rate estimation, which can be specified using ‘$’ on branches in the provided tree file. For instance, (((taxon1$1: 0.01, taxon2$1: 0.01)$3: 0.01, taxon3$2: 0.02)$3: 0.01, taxon4$2: 0.03); specifies three rates, one for the branches leading to taxon1 and taxon2 ($1), one for the branches leading to taxon3 and taxon4 ($2), and one for the remaining branches ($3). The modified tree files are no longer in the conventional Newick format, we have developed a function read.tree2 in DiscML to read such modified tree files.

### Additional features

DiscML allows binary (1s/0s) analysis on data with more than two character states by converting all non-zero characters to 1s with simplify=TRUE.

## Results and discussion

**Figure 1:**
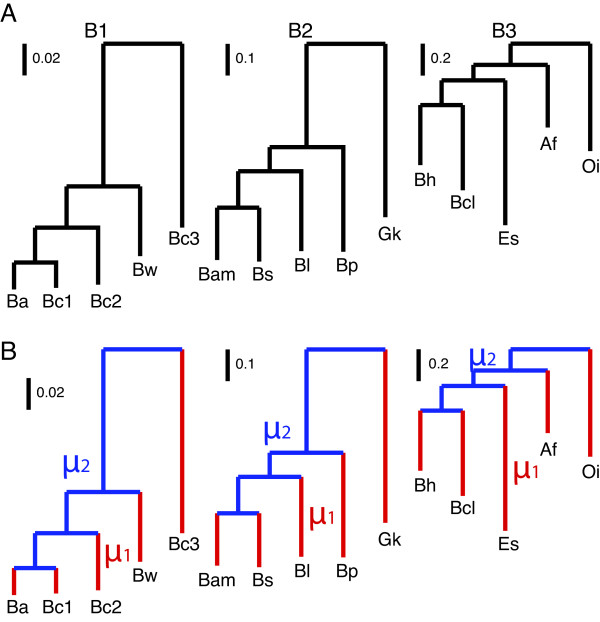
**Phylogenetic relationship of three Bacillaceae (B1, B2, B3) clades, on which the evolutionary rates of gene families are estimated using DiscML.** **A**, a constant rate is estimated on each phylogeny; **B**, separate rates are estimated for external branches ( *μ*_1_) versus internal branches ( *μ*_2_) on each phylogeny. These three clades were studied in our previous study on gene presence, absence, and fragments [20]. Gene families are recategorized, with gene absence and fragments as character state 0, single-copy genes as 1, and gene families with two or more members as 2.

DiscML was first tested using the gene family data on three Bacillaceae clades (Figure 1A, Additional file 1 and [20]). In the previous study [20], we distinguished gene fragments from gene absence and gene presence. In this study, we eliminated the character state specific for gene fragments and re-categorized gene fragments as gene absence or character state 0, single-copy genes as character state 1, and gene families with two or more members as 2 (Additional file 1), so that the application of BD models on these data is meaningful. It is worth to note that, though the number of character states is restricted to three here, DiscML is flexible and capable of analyzing a large number of character states.

The performance of DiscML is found to be reliable. For instance, the ER+0 model with the option of simplify=TRUE in Table 1 is mathematically identical to the *M*_00_ model in [20]. The optimization in [20] was achieved using the Nelder-Mead simplex method [25], while the optimization in Table 1 was achieved using the PORT routines [17]. Importantly, the DiscML estimates are identical to the previous estimates for all three clades. As expected, the parameter-rich models consistently outperformed the nested simplistic models (e.g., Ln*L*BDARD > Ln*L*BDISYM > Ln*L*BDER; Ln*L*ARD > Ln*L*SYM > Ln*L*ER). Consistent with previous studies [3, 20, 26], rate estimates in closely related clades tend to be higher than those in distantly related clades due to the transient nature of many acquired genes (Table 1). Tested on an Intel Core i7 (3.4 Ghz) 16 GB RAM Dell desktop, the computation using DiscML is fast (Table 2). For instance, the ER (1s/0s only) analysis took 49 seconds (0 m 49 s) for B1 (5453 gene families), 60 seconds (1 m 00 s) for B2 (5614 gene families), and 86 seconds (1 m 26 s) for B3 (6813 gene families). Computational time increases with the complexity of transition rate matrices and the addition of estimated parameters. For instance, the ER+0+ *π* + *Γ* analysis took 82 m 22 s for B1, 81 m 20 s for B2, and 178 m27 s for B3 (Table 2).

**Table 2:** **Computational time on an Intel Core i7 (3.4 Ghz) 16 GB RAM Dell desktop to generate the results in Table** 1

Models	B1(5453)	B2(5614)	B3(6813)
ER (1s/0s only)	0 m 49 s	1 m 00 s	1 m 26 s
ER+0 (1s/0s only)	1 m 39 s	2 m 01 s	3 m 03 s
BDER	0 m 48 s	1 m 06 s	1 m 36 s
BDISYM	1 m 58 s	2 m 20 s	3 m 01 s
BDARD	7 m 54 s	6 m 58 s	8 m 28 s
ER	1 m 04 s	1 m 15 s	1 m 17 s
SYM	3 m 14 s	4 m 47 s	5 m 31 s
ARD	9 m 53 s	9 m 12 s	16 m 59 s
GTR(SYM+ *π* ^REV^)	9 m 04 s	9 m 54 s	11 m 44 s
ER+0	1 m 36 s	2 m 34 s	2 m 21 s
ER+ *π*	2 m 41 s	3 m 13 s	4 m 40 s
ER+ *Γ*	12 m 00 s	39 m 01 s	45 m 23 s
ER+0+ *π* + *Γ*	82 m 22 s	81 m 20 s	178 m 27 s
ER+0+ *π* ^REV^ + *Γ*	80 m 13 s	67 m 33 s	91 m 42 s

The number of gene families is shown in parentheses for each clade. The time is shown in minutes (m) and seconds (s).

DiscML was developed to allow separate rates among branches since evolutionary rates can vary among lineages [27–29]. In the three Bacillaceae clades, we assigned separate rates between external branches ( *μ*_1_) and internal branches ( *μ*_2_) as illustrated in Figure 1B. Our results in Table 3 support the previous findings of higher gene turnover rates on external branches than those on internal branches [26, 30].

**Table 3:** **Separate rates on branches estimated from the gene family data in the Bacillaceae (B1, B2, B3) clades**

Models	Parameters	B1	B2	B3
( *μ*_1_=*μ*_2_)ER	*μ*	2.940	0.638	0.459
	Ln*L*	-21411	-23273	-31405
( *μ*_1_≠*μ*_2_)ER	*μ*_1_	4.430	0.674	0.477
	*μ*_2_	0.306	0.526	0.344
	Ln*L*	-21045	-23267	-31395
	2 *Δ*Ln*L*	732 ^∗∗∗^	14 ^∗∗∗^	20 ^∗∗∗^

*μ*_1_is for external branches, while *μ*_2_ is for internal branches on each tree as illustrated in Figure 1B.

^∗∗∗^ *P*<0.001 (df=1), as 2 *Δ*Ln*L* approximately follows a *χ* ^2^-distribution.

**Figure 2:**
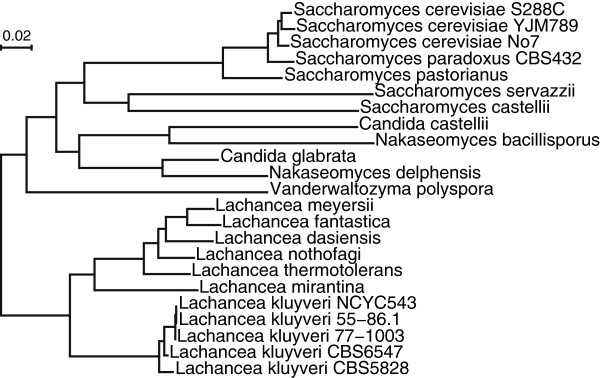
**Phylogenetic relationship of the yeast strains in the** ***Saccharomyces*** **complex, on which the rates of mitochondrial intron gain and loss are estimated using DiscML.** The phylogeny was reconstructed using the concatenated sequences of all mitochondrial protein genes after excluding the *var1* gene.

**Figure 3:**
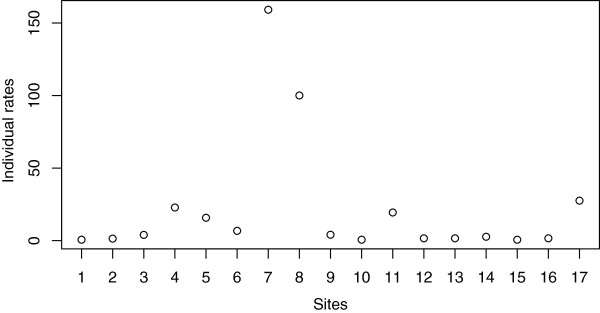
**Plot of individual turnover rates of the 17 mitochondrial introns in yeast.** Ten introns in the *cox1* gene are shown as sites 1-10, six introns in the *cob* gene are shown as sites 11-16, and one intron in the 21S rRNA gene is shown as site 17.

It is often of interest for users to know the individual rate of each character site. Previously, we have shown that the mitochondrial intron in the 21S rRNA gene undergoes very rapid turnover in yeast [31]. In this study, we estimated the individual rates of all 17 mitochondrial introns on the yeast phylogeny (Figure 2 and Additional file 1) based on the intron distribution pattern (Additional file 1). On the plot generated by DiscML using ind=TRUE (Figure 3), users can visually compare the individual rates of different introns. For instance, the introns at sites 7 and 8 have faster turnover rates than the 21S rRNA intron at site 17 (Figure 3). The R commands used in the study are provided in Additional file 1.

## Conclusion

We illustrated the versatility of DiscML on different types of data and analyses. With a great flexibility and fast computational speed, we are confident that DiscML can be used in a variety of studies on different discrete characters.

## Availability and requirements

**Project name:** DiscML

**Project home page:**http://cran.r-project.org/web/packages/DiscML/index.html

**Operating system(s):** Platform independent.

**Programming language:** R.

**Other requirements:** R (2.14 or newer); R-package: ape from CRAN.

**License:** GNU GPL

## Electronic supplementary material

Additional file 1: **Files and commands used in the analyses of the B1 clade and the yeast clade.** B1.tre is the B1 tree in the conventional Newick format. B1_pattern contains the distribution pattern of gene families in the B1 clade with gene absence and fragments as 0s, single-copy genes as 1s, and gene families with two or more members as 2s. Each column is for one genome, and each row is for one gene family. B1_2rates.tre is the B1 tree with assigned separate rates for external branches and internal branches. The rate for external branches is $1, and the rate for internal branches is $2. The yeast.tre file is the phylogenetic tree of 13 yeast strains in the conventional Newick format. The intron_pattern file contains the distribution pattern of the 17 mitochondrial introns in the 13 yeast strains. Each column is for one intron, and each row is for one strain. Data matrix in this format will need to be transformed before the analysis (see R.inputs for details). Some R commands are in R.inputs. (ZIP 6 KB)
